# Prevalence of genital *Mycoplasma* in pregnancies with shortened cervix

**DOI:** 10.1007/s00404-023-07252-w

**Published:** 2023-10-24

**Authors:** Maximilian Rauh, Franziska Werle, Börge Schmidt, Christian Litzka, Maria Emilia Solano, Angela Köninger

**Affiliations:** 1 University Department of Obstetrics and Gynecology, Clinic St. Hedwig of The Order of St. John, University of Regensburg, Steinmetzstr. 1-3, D-93049 Regensburg, Germany; 2 Institute for Medical Informatics, Biometry and Epidemiology (IMIBE), University Hospital of Essen, Hufelandstraße 55, D-45147 Essen, Germany; 3 Laboratory of Translational Peronatology, University of Regensburg, Biopark 1-3, D-93053 Regensburg, Germany

**Keywords:** *Mycoplasma*, *Ureaplasma*, Cervical insufficiency, Preterm birth

## Abstract

**Objective:**

To determine whether colonisation with genital *Mycoplasma* species (spp.) in patients presenting with a shortened cervix before 34th week of pregnancy is associated with preterm birth.

**Methods:**

The collection of this retrospective study consisted of 100 pregnant women who presented to a German Tertiary Perinatal Center between 2017 and 2020 due to a shortened cervix defined as a cervical length of 25 mm or shorter measured by transvaginal ultrasound before 34 weeks of gestation. At the time of admission, gestational age ranged from 18 + 4 to 33 + 3 weeks (+ days) of pregnancy. All patients underwent urine polymerase chain reaction (PCR) for genital *Mycoplasma* [*Ureaplasma* (*U*.) *urealyticum*, *U*. *parvum*, *M*. *hominis* or *M*. *genitalium*]. Patients who were tested positive underwent a therapy with macrolides (azithromycin or clarithromycin).

**Results:**

37% of the patients were positive for *Ureaplasma* spp., whereas 5% (5 patients) were *Mycoplasma* spp.-positive. All the latter were simultaneously colonised with *Ureaplasma* spp. *Ureaplasma*-positive patients were significantly younger than those who were tested negative. Median maternal age at examination was 30 years (a) versus 31a (*p* = 0.04). There was no difference between *Ureaplasma*-positive and -negative patients regarding median maternal body mass index (BMI) (kg/m^2^) (23.4 versus 22.3, *p* = 0.41), cervical length at admission (mm) (15 versus 17, *p* = 0.17), gestational age at examination (days, d) (198 versus 197, *p* = 0.97) or gestational age at birth (d) (250 versus 257, *p* = 0.33), respectively. Comparing *U*. *parvum*-positive and *U*. *urealyticum*-positive patients, there was some weak indication that *U*. *parvum*-positive patients may get a shortening of the cervix earlier in pregnancy, as the median gestational age at examination was 196d versus 215d (*p* = 0.06). Regarding *Mycoplasma*-positive and -negative patients, there was no difference in all examined parameters.

**Conclusions:**

Overall, one-third of all women in our study with a shortened cervix before 34th week of pregnancy were colonised with genital *Mycoplasma* spp. We were able to show that pregnant women, who were treated with antibiotics when tested positive for genital *Mycoplasma*, gave birth at the same gestational age as patients with a shortened cervix without detected *Mycoplasma*. This raises the question of whether routine testing and early antibiotic treatment should be established in prenatal care.

## What does this study add to the clinical work

**Table:** 

We were able to show that colonization with genital Mycoplasma is not associated with an earlier onset of threatening preterm birth nor with a higher degree of cervical shortening. Additionally, our results show that pregnant women, who are treated with antibiotics due to colonization with genital mycoplasma, gave birth at the same gestational age as patients with a shortened cervix without detection of mycoplasma.	

## Introduction

### What does this study add to the clinical work?

We were able to show that colonisation with genital *Mycoplasma* is not associated with an earlier onset of threatening preterm birth nor with a higher degree of cervical shortening. Additionally, our results show that pregnant women, who are treated with antibiotics due to colonisation with genital *Mycoplasma*, gave birth at the same gestational age as patients with a shortened cervix without detection of *Mycoplasma*.

Genital infections are of great importance in obstetric medicines as they are a main cause of premature birth [35]. Reasons for preterm delivery can be divided into spontaneous onset of labour, preterm birth after preterm premature rupture of membranes (PPROM) and maternal or foetal complications, such as preeclampsia [14] that results in iatrogenic preterm birth. Premature birth caused by spontaneous contractions with and without PPROM is often caused by ascending genital infections and therefore can be controlled by antibiotic treatment [15]. This is of high relevance since the global preterm birth rate remains high: in 2014, 10.6% of all babies were born preterm worldwide, resulting in approximately 15 million preterm births [6]. Globally, complications due to prematurity account for 14% of infant mortality [26]. There is an inverse relationship between gestational age at birth and hospital costs during the neonatal period [30] as well as the cumulative costs of hospital inpatient admissions in the first 10 years [31]. Since patients born preterm suffer from an increased all-cause morbidity including respiratory, cardiovascular, endocrine and neurological diseases, the consequences of a premature birth persist until adulthood [8]. A reduction in the rate of premature births is therefore of high interest not only for the affected individuals and their families, but also for public health systems.

The strategy to reduce premature births mainly includes the treatment of genital infections. *Mycoplasma* and *Ureaplasma* species (spp.) are amongst the most common pathogens detected in the urogenital tract regardless of whether symptoms are present or not [20, 32, 39]. Their presence in the vagina is influenced by age, hormones, sexual activity and pregnancy [39].

Often summarised under the term “genital mycoplasma”, *Mycoplasma* and *Ureaplasma* are two genus of the family Mycoplasmataceae, order Mycoplasmatales, class Mollicutes. The term “mollicutes” derives from the Latin expression for soft “mollus”. They are considered the smallest independently surviving bacteria. Due to their small genome, they are unable to produce a stable cell wall which results in a pleomorphic shape. Their metabolic performance is also restricted and depends on a parasitic or saprophytic nutrition [36, 38]. The metabolism differs amongst the species: *M*. *genitalium* metabolises glucose, *M*. *hominis* arginine and *U*. *urealyticum* urea [12]. Through adherence to host cells, genital *Mycoplasma* can interfere with the host metabolism. They can activate macrophages and monocytes and lead to the secretion of inflammatory cytokines (tumour necrosis factor α, interferon γ and interleukin-1, -6, -8, -12, -16), thereby causing cell injury [37]. The ability to modify proteins in their membranes makes it possible for *Mycoplasma* to evade the host’s immune response [37]. *Mycoplasma* are normally found extracellularly on the mucosa of the respiratory and genital tract. The genitourinary tract acts as the primary site of colonisation of *M*. *hominis*, *M*. *genitalium*, *U*. *urealyticum* and *U*. *parvum* [36, 38].

Since the role of genital *Mycoplasma* colonisation in pregnancy is discussed controversially, the screening of asymptomatic or symptomatic individuals is yet not recommended [19]. Some authors see no association between colonisation with *M*. *genitalium* and adverse pregnancy outcomes in general [24], between the prevalence of *M*. *hominis*, *U*. *parvum* or *U*. *urealyticum* and a preterm or full-term delivery [17] or between the prevalence of *U*. *urealyticum* and *M*. *hominis* in patients who developed preterm labour with or without resulting in preterm delivery [7].

The majority of authors however reported opposite results: colonisation with *M*. *genitalium* [11] and *M*. *hominis* [9] is independently associated with spontaneous preterm delivery,colonisation with *Ureaplasma* spp. is associated with PPROM in women with signs of preterm delivery [2] and colonisation with *Ureaplasma* spp. is significantly correlated with preterm delivery especially in combination with an abnormal vaginal flora [4].

Even when co-existing infections are excluded, colonisation with *U*. *urealyticum* is associated with a significant decrease of gestational age and birth weight as well as a significant increase in the rate of chorioamnionitis [1]. In fact, cervical colonisation with *U*. *urealyticum* is not just only associated with preterm birth, but also with chorioamnionitis [27]. *Ureaplasma* spp. can frequently be isolated from preterm placentas < 32 weeks of pregnancy [28]. Together with the placenta, cord blood cultures of nearly one of every four preterm neonates (23–32 weeks) result positive for *M*. *hominis* or *U*. *urealyticum* [13]. *U. urealyticum*, *U. parvum* and *M. hominis* can cause a severe intra-amniotic and maternal inflammatory response [29].

However, the mere colonisation of maternal amniotic fluid with genital *Mycoplasma* (detected by amniocentesis due to other medical reasons in the first trimester in asymptomatic pregnant women) is not associated with an unfavourable pregnancy outcome [23].

Reasons why these weakly pathogenic organisms can lead to preterm birth are still unclear and need further investigation. One possible reason could rely on the fact that high maternal progesterone and oestrogen levels stimulate genital *Mycoplasma* growth [12] or modulate the host immune system, which plays a critical role in ascending genital infections [4]. Furthermore, a maturation of the intrauterine foetal immune system might cause an increased sensitivity to weakly pathogenic bacteria in the second trimester [28].

These conflicting statements and the gaps in understanding about the pathogenicity of genital *Mycoplasma* underscore the need for further research. Therefore, in this study, we aim to analyse the frequency of genital *Mycoplasma* spp. colonisation in patients presenting with a shortened cervix before 34th week of pregnancy resulting in preterm or term birth, respectively.

## Materials and methods

The present retrospective study includes 100 pregnant women who presented to a German Tertiary Perinatal Center before 34 weeks of gestation due to shortened cervix, defined as a cervical length of 25 mm or shorter as determined by transvaginal ultrasound. Voluson S8 and an E8 ultrasound machines were used (GE Healthcare GmbH, Solingen, Germany). Patients presenting to the Perinatal Center between January 2017 and December 2020 were selected consecutively.

All patients underwent a urine polymerase chain reaction (PCR) for genital *Mycoplasma* spp. (*U*. *urealyticum*, *U*. *parvum*, *M*. *hominis* or *M*. *genitalium*). For this purpose, midstream urine was analysed at the laboratory of the Department of Microbiology and Hygiene of the University of Regensburg. Patients who tested positive underwent a therapy with macrolides (azithromycin or clarithromycin) according to the physician´s actual opinion including current standard of the clinic and patient´s comorbidity (Table 1).

**Table 1:** Therapy after diagnosis of genital *Mycoplasma*

Therapy (duration)	Genital *Mycoplasma*-positive (37 women)	
	*n*	(%)
Clarithromycin 250 mg 1–0–1 (10d)	16	43.2
Clarithromycin 500 mg 1–0–1 (7d)	1	2.7
Azithromycin 1 g (once)	8	21.6
Azithromycin 1.5 g (once)	5	13.5
Birth before antibiotic treatment	5	13.5
Discharge before antibiotic treatment	2	5.4

*d* days; antibiotics were administered orally

The study was approved by the ethics committee of the University of Regensburg (No. 21-2526-104). Patient data collected during the inpatient treatment of the participants were retrieved from a digital archive of the clinic system (Viewpoint 5.0, Viewpoint 6.0; SAP). The parameters collected were maternal age in years (a), body mass index (BMI in kg/m^2^), cervical length in millimetre (mm), gestational age at examination time and gestational age at birth in days (d).

For statistical analyses, *t*-test (normally distributed parameters), Mann–Whitney *U *test (non-parametrical parameters) as well as Chi-square-test (grouped data) were used to compare groups (SigmaPlot 14.0, Inpixon GmbH, Düsseldorf, Germany.) *P* < 0.05 was considered significant.

## Results

The analysis of 100 patients with a shortened cervix revealed that 37% (37/100) were positive for *Ureaplasma* spp. Amongst them, 11% were positive for *U*. *urealyticum* (4/37), 78% were positive for *U*. *parvum* (29/37) and in 11% the species could not be differentiated (4/37). There was no case of infection with two serovars at the same time.

Only 5% (5/100) amongst all analysed patients were *Mycoplasma* spp.-positive. All of them were colonised with *M. hominis* (5/5), whereas 20% (1/5) were additionally positive for *M*. *genitalis*. There was no patient who was colonised with *Mycoplasma* spp. only, since all of them were simultaneously colonised with *Ureaplasma* spp.

81% (30/37) of genital *Mycoplasma*-positive patients received antibiotic treatment, which were administered orally. 45.9% received Clarithromycin and 35.1% (13/37) received Azithromycin.

13.5% (5/37) gave birth before the test results were available. Therefore, therapy was not yet applied. 5.4% (2/37) were discharged with stable cervix length before antibiotic therapy was started (Table 1).

Concerning the whole study group, the median age in years (a) was 30 a [IQR (interquartile range) 28–34] and median BMI was 22.7 kg/m^2^ [IQR 20.0–26.5]. The median cervical length in millimetre (mm) was 15 [IQR 11–20]. At the time of examination, median gestational age in days (d) was 198 d [IQR 179–221], whilst the median gestational age at birth was 257 d [IQR 231–274] (Table 2).

**Table 2:** Patient characteristics of the study cohort (*n* = 100)

Maternal age (a)	mean (STD)	31 (5.49)
	Median (IQR)	30 (28–34)
Maternal BMI (kg/m^2^)	Mean (STD)	23.7 (4.95)
	Median (IQR)	22.7 (20.0–26.5)
Cervical length (mm)	Mean (STD)	15 (5.77)
	Median (IQR)	15 (11–20)
Gestational age at examination (d)	Mean (STD)	197 (25.7)
	Median (IQR)	198 (179–221)
Gestational age at birth (d)	Mean (STD)	248 (30.46)
	Median (IQR)	257 (231–274)

*a* years, *kg/m*^*2*^ kilogramme/square metres, *mm* millimetre, *d* days, *IQR* interquartile rate, *STD* standard deviation

At the time of diagnosis of the shortened cervix, 46% (17/37) of the patients positive for *Ureaplasma* spp. were younger than 30 years (a) (8.1% ≤ 19; 10.8% 20–24; 27.0% 25–29) and 54% (20/37) were at least 30a old (40.5% 30–34; 10.8% 35–39; 2.7% ≥ 40). In the group of patients that were simultaneously positive for *Mycoplasma* spp. 60% (3/5), the majority of women were younger than 30a (20% ≤ 19; 20% 20–24; 20% 25–29) and 40% (2/5) at least 30a old (40% 30–34) (Table 3).

**Table 3:** Comparison of *Ureaplasma* spp. and *Mycoplasma* spp. colonization with maternal age *(a* = *years)* at time of diagnosis of a shortened cervix; all *Mycoplasma* spp. positive patient had a co-colonisation with *Ureaplasma* spp.

Maternal age (a)	*Ureaplasma* spp.-positive (37 women)		*Mycoplasma* spp.-positive (5 women)	
	*n*	(%)	*n*	(%)
≤ 19	3	8.1	1	20
20–24	4	10.8	1	20
25–29	10	27.0	1	20
30–34	15	40.5	2	40
35–39	4	10.8	0	0
≥ 40	1	2.7	0	0

**Fig. 1:**
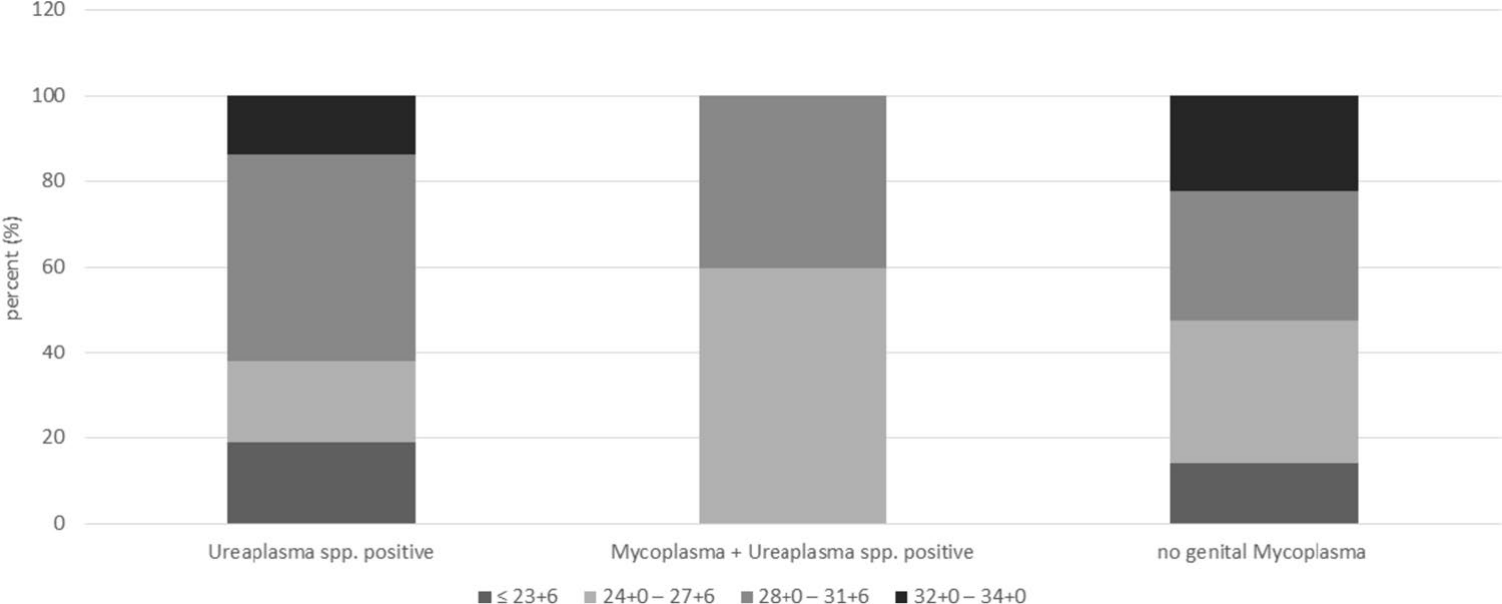
Distribution of gestational age groups at admission of women with shortened cervix

As shown in Fig. 1, the percentage of patients presenting before the 28th week of gestation is comparable between the different colonisation subgroups.

*Ureaplasma*-positive patients were significantly younger than those tested negative. Median maternal age at examination was 30a [IQR 26–32] versus 31a [IQR 29–35] (*p* = 0.04). There was no difference between *Ureaplasma*-positive and -negative patients regarding median maternal BMI (kg/m^2^): 23.4 kg/m^2^ [IQR 20.7–26.6] versus 22.3 kg/m^2^ [IQR 19.7–26.2], (*p* = 0.41), cervical length: 15 mm [IQR 10–20] versus 17 mm [IQR 12–21], (*p* = 0.17), gestational age at examination: 198d [IQR 171–220] versus 197d [IQR 181–223], (*p* = 0.97] or gestational age at birth: 250d [IQR 229–270] versus 257d [IQR 234–277], (*p* = 0.33) (Table 4), respectively.

**Table 4:** Analysed parameters in *Ureaplasma*-positive and -negative patients

		*Ureaplasma*-positive (*n* = 37)	*Ureaplasma*-negative (*n* = 63)	*p*-value
Maternal age (a)	Mean (STD)	29 (5.89)	31(5.11)	**0.04**
	Median (IQR)	30 (26–32)	31 (29–35)	
Maternal BMI (kg/m^2^)	Mean (STD)	23.3 (5.39)	23.3 (4.67)	0.41
	Median (IQR)	23.4 (20.7–26.6)	22.3 (19.7–26.2)	
Cervical length (mm)	Mean (STD)	14 (5.96)	16 (5.60)	0.17
	Median (IQR)	15 (10–20)	17 (12–21)	
Gestational age at examination (d)	Mean (STD)	197 (25.11)	197 (26.25)	0.97
	Median (IQR)	198 (171–220)	197 (181–223)	
Gestational age at birth (d)	Mean (STD)	246 (29.17)	250 (31.34)	0.33
	Median (IQR)	250 (229–270)	257 (234–277)	

*a* years, *kg/m*^*2*^ kilogramme/square metres, *mm* millimetre, *d* days, *IQR* interquartile rate, *STD* standard deviation

Comparing *U*. *parvum*-positive and *U*. *urealyticum*-positive patients, there was a tendency that *U*. *parvum*-positive patients develop a shortening of the cervix earlier in pregnancy: median gestational age at presentation was 196d [IQR 168–218] versus 215d [IQR 180–222], (*p* = 0.06). However, the median gestational age at birth did not differ in both groups: 244d [IQR 229–269] versus 235d [IQR 205–269], (*p* = 0.11). No differences were found with regard to maternal age: 29 a [IQR 26–33] versus 31a [IQR 29–31], (*p* = 0.51), maternal BMI: 23.6 kg/m^2^ [IQR 21.2–28.1^2^] versus 23.3 kg/m^2^ [IQR 19.4–25.1], (*p* = 0.53) and cervical length: 12 mm [IQR 9–19] versus 18 mm [IQR 7–22] (*p* = 0.37) at admission (Table 5).

**Table 5:** Analysed parameters in patients positive for *Ureaplasma* *parvum* and *urealyticum*

		*U*. *parvum*-positive (*n* = 29)	*U*. *urealyticum*-positive (*n* = 4)	*p*-value
Maternal age (a)	Mean (STD)	29 (6.38)	30 (0.96)	0.51 ^∆^
	Median (IQR)	29 (26–33)	31 (29–31)	
Maternal BMI (kg/m^2^)	Mean (STD)	25.0 (5.71)	22.6 (3.08)	0.53 ^∆^
	Median (IQR)	23.6 (21.2–28.1)	23.3 (19.4–25.1)	
Cervical length (mm)	Mean (STD)	13 (5.88)	15 (8.30)	0.37 *
	Median (IQR)	12 (9–19)	18 (7–22)	
Gestational age at examination (d)	Mean (STD)	195 (26.7)	206 (24.24)	0.06 *
	Median (IQR)	196 (168–218)	215 (180–222)	
Gestational age at birth (d)	Mean (STD)	244 (29.91)	236 (33.10)	0.11*
	Median (IQR)	244 (229–269)	235 (205–269)	

*(a* years, kg/m^2^ kilogramme/square metres, *mm* millimetre, *d* days, *IQR* inter-quartile rate, *STD* standard deviation)

^∆^Shapiro–Wilk test, *Mann–Whitney test

Finally, no significant differences were observed when comparing the parameters between patients simultaneously colonised with *Mycoplasma* spp. and those who had no colonisation with *Mycoplasma* spp. Median maternal age: 28a [IQR 20–33] versus 30a [IQR 29-3a], (*p* = 0.18), the cervical length: 15 mm versus 15 mm [14-20] [IQR 11–20], (*p* = 0.79), the gestational age at examination: 195d [IQR 173–217] versus 198d [IQR 180–221], (*p* = 0.69), and the gestational age at birth: 260d [IQR 223–270] versus 257d [IQR 231–274], (*p* = 0.89] remained unchanged (Table 6). A trend towards a lower BMI in *Mycoplasma*-positive compared to negative patients (18.7 kg/m^2^ [IQR 17.5–23.1] versus 22.9 kg/m^2^ [IQR 20.2–26.9], (*p* = 0.07)) was observed.

**Table 6:** Analysed parameters in *Mycoplasma*-positive and -negative patients, all *Mycoplasma* spp.-positive patient had a co-colonisation with *Ureaplasma* spp.

		*Mycoplasma*-positive (*n* = 5)	*Mycoplasma*-negative (*n* = 95)	*p*-value
Maternal age (a)	Mean (STD)	27 (6.73)	31 (5.37)	0.18
	Median (IQR)	28 (20–33)	30 (29–34)	
Maternal BMI (kg/m^2^)	Mean (STD)	20.0 (3.00)	23.9 (4.96)	0.07
	Median (IQR)	18.7 (17.5–23.1)	22.9 (20.2–26.6)	
Cervical length (mm)	Mean (STD)	16 (3.42)	15 (5.87)	0.79
	Median (IQR)	15 (14–20)	15 (11–20)	
Gestational age at examination (d)	Mean (STD)	195 (22.07)	197 (25.98)	0.69
	Median (IQR)	195 (173–217)	198 (180–221)	
Gestational age at birth (d)	Mean (STD)	249 (26.79)	248 (30.77)	0.89
	Median (IQR)	260 (223–270)	257 (231–274)	

*a* years, *kg/m*^*2*^ kilogramme/square metres, *mm* millimetre, *d* days, *IQR* inter-quartile rate, *STD* standard deviation

## Discussion

In this study, we were able to demonstrate that there was no difference in gestational age at admission because of a shortened cervix between patients colonised with genital *Mycoplasma* and those who were negative. We were able to show that women who were treated with macrolides after positivity for genital *Mycoplasma* in the urine PCR gave birth at a gestational age comparable to that of patients without evidence of colonisation.

In the present study, a cervical length of 25 mm or less was defined as shortened cervix. This is in accordance with obstetric standards, which is based on the knowledge that women with a cervical length of 25 mm or less have a significantly shorter duration of pregnancy compared to women with a longer cervix [21].

Our results concerning the prevalence of genital *Mycoplasma* in the studied population are comparable with those reported in the literature. Prevalence varies greatly depending on the patient population, the sample site and the test system used: Bayraktar et al. report a positivity rate of 44% for U. urealyticum, 4% for M. hominis and 6% for both species in the cultures from endocervical swabs taken from a group of 50 symptomatic pregnant women in Turkey [3]. Choi et al. report detection rates of 62.7% for U. urealyticum, 12.7% for *M. hominis* and 0% for *M*. *genitalium* in the vaginal swabs by PCR amongst 126 women who suffered from preterm labour with and without spontaneous preterm birth in Korea [7]. Mitsunari et al. report a rate of 85% positive for *U. urealyticum* DNA in the PCR of cervical swabs of 23 women with preterm labour and delivery in Japan [27]. In a prospective Spanish study including 200 pregnant women with preterm labour and intact membranes, 51.5% endocervical cultures were positive for *U. urealyticum*, but 0% for *M. hominis* [27]. Of note, these studies did not differentiate between *U. urealyticum* and *U. parvum*, limiting the comparability to our results. This is because the designation *U. urealyticum* biovar 1 and 2 was treated commonly at the beginning of the determination era. Since 2002, the distinction between *U. urealyticum* and *U. parvum* was established due to the finding of distinct phenotypic and genotypic properties [34].

To our knowledge, there exists no further study on pregnant women with threatened preterm birth in the second trimester with a differentiation between genital colonisation with U. urealyticum und U. parvum as possible causative bacteria. However, in a study with 4330 asymptomatic pregnant women, the influence of these two species on preterm birth was analysed [33]. The PCR analysis of genital swabs collected between 12th and 14th week of pregnancy revealed that 37% of the women were positive for *U. parvum*, 5.9% for *U. urealyticum* and 3.1% for both species. Genital colonisation with *U. parvum* but not with *U. urealyticum* was significantly associated with spontaneous preterm birth [33].

Similarly, a study of 877 women examined before the 11th week of pregnancy for *Ureaplasma* and *Mycoplasma* using vaginal swabs showed that colonisation with *U. parvum* was associated with premature birth and late miscarriage in contrast with U. urealyticum [22].

Although these two studies were unable to show associations between the colonisation with U. urealyticum and premature birth, the increased risk for late miscarriages in the case of colonisation with *U. parvum* could indicate that pregnant women may develop obstetric problems earlier in pregnancy. This is in line with our finding that women with *U. parvum* colonisation tend to develop a shortening of the cervix—important risk factor for late miscarriage—at an earlier gestational age.

Women with a shortened cervix and evidence of genital *Mycoplasma* have the same obstetric outcome after a standardised therapy as women with cervical shortening without evidence of genital *Mycoplasma* colonisation. This corresponds to the result of a study by Vouga et al. In a retrospective analysis, including data of 5377 pregnant women with imminent preterm birth in the 25–37th week of gestation, 2259 women (42%) were tested positive for *Mycoplasma* in a genital swab and received therapy with clindamycin (4 × 150 mg, 5 days). The preterm birth rate was 44.1% in uncolonized women and even lower in *Ureaplasma*-positive (40.9%) and *Mycoplasma*-positive (37.7%) women after therapy [40]. These results allow the conclusion that antibiotic treatment of the colonisation with genital *Mycoplasma* improves the obstetric outcome compared to women whose cervical shortening is not associated with a colonisation.

## Strength and limitations

Our study has strength and limitations. The latter includes the retrospective study design, which was carried out at a single institution. A point of criticism might also be the lack of adjustment for colonisation and treatment of co-infections and therefore cannot assess a potential effect on cervical shortening caused by other agents. In the literature, the influence of co-infection is discussed controversially. Rittenschober-Böhm et al. found in a multivariable analysis on data of 4330 pregnant women a stronger association with preterm birth when both *U*. *parvum* and bacterial vaginosis (BV) were present rather than only one of them [33].

This corresponds to the results of study published by Breugelmans et al., which examined the vaginal flora of 1998 pregnant women according to the criteria for diagnosis of bacterial vaginosis in the first trimester and additionally performed a culture for *Ureaplasma* spp. A logistic regression analysis showed that preterm delivery was correlated with the presence of *Ureaplasma* spp. The risk increased in cases of both *Ureaplasma* colonisation and abnormal vaginal flora [4]. In contrast, a further study stated that the degree of colonisation with *U*. *urealyticum* correlates strongly with an adverse effect on pregnancy outcome even when a co-existing infection is excluded. A multivariate analysis was performed on data of 172 pregnant women with genital colonisation with *U*. *urealyticum* without co-infection and 123 women with negative cultures for *U*. *ur*ealyticum [1].

Furthermore, we did not opt for the most sensitive method for detecting genital *Mycoplasma*: Lillis et al. compared detection rates via PCR in first-void urine, vaginal, cervical and rectal swabs for *M*. *genitalium* in a study involving 400 women. 17.5% (*n* = 70) of them were tested positive. The best single test method was the vaginal swab (85.7%, 60/70) followed by endocervical swab (74.3%, 52/70), urine (61.4%, 43/70), and rectal swab (24.2%, 17/70). Therefore, an endocervical swab dipped into the vaginal secret might result in the highest detection rate [25]. In a study of 1110 women who underwent a screening for sexual transmitted infections, a genital swab resulted in a significantly higher sensitivity rate in detecting *M*. *genitalium* via PCR compared with first-void urine (75% vs 96%, *p* = 0.001) [18]. However, since we performed identical methods in all patients, this limitation may not affect our results finally.

Regarding the detection methods, culture is still considered as the gold standard because it allows testing the antimicrobial susceptibility. However, disadvantages of this method are their longer duration, more complex culture process and higher costs compared to PCR. In addition, the limit of detection is lower with PCR diagnostic [5]. Thus, in the case of cervical shortening, as in the present study, PCR testing resulted in faster results, which is an important aspect in the view of threatening preterm birth.

An additional point of criticism might be the difference in therapy depending on the results of the urine PCR. However, this is a retrospective intention-to-treat study that rather aims to analyse the effect of a therapy but focuses on the prevalence of Myco- and *Ureaplasma* in women with a shortened cervix.

There was no patient in our study who was colonised with *Mycoplasma* spp. alone. All *Mycoplasma* spp.-positive patients had a co-colonisation with *Ureaplasma* spp. Thus, no statement can be made about the effects of sole colonisation with *Mycoplasma* spp.

Overall, one-third of the women included in our study with cervical shortening were colonised with genital *Mycoplasma*. As the main result of our study, we were able to show that colonisation is not associated with an earlier onset of threatening preterm birth nor with a higher degree of cervical shortening. Additionally, our results show that pregnant women, who are treated with antibiotics due to colonisation with genital *Mycoplasma*, gave birth at the same gestational age as patients with a shortened cervix without detection of *Mycoplasma*. Further research now is recommended to explore the effect of antibiotic therapy to know more about the final effect of Myco- and Ureaplasma colonization on preterm birth.

## Data Availability

Data are available upon request.
